# The effect of childhood trauma on moral cognition in patients with schizophrenia

**DOI:** 10.3389/fpsyt.2024.1432407

**Published:** 2024-09-30

**Authors:** Xing Peng, Yu-shen Ding, Bo Ren, Xi-xi Zhao, Fei-fei Wang, Jie Zhao, Yuan-yuan Zhang, Xiu-jun Zhang, Fu-chun Zhou, Chuan-yue Wang

**Affiliations:** ^1^ School of Public Health, North China University of Science and Technology, Tangshan, China; ^2^ Beijing Key Laboratory of Mental Disorders, National Clinical Research Center for Mental Disorders & National Center for Mental Disorders, Beijing Anding Hospital, Capital Medical University, Beijing, China; ^3^ Advanced Innovation Center for Human Brain Protection, Capital Medical University, Beijing, China; ^4^ State Key Laboratory of Cognitive Neuroscience and Learning and IDG/McGovern Institute for Brain Research, Beijing Normal University, Beijing, China; ^5^ China Rehabilitation Research Center, Department of Psychiatry, Beijing, China; ^6^ Department of Psychiatry, Jingda Psychiatric Hospital, Hengshui, China

**Keywords:** childhood trauma, moral cognition, schizophrenia, CTQ, MFQ

## Abstract

**Objective:**

The aim of this study was to investigate whether a potential moral cognitive impairment (failure in understanding moral rules) exists in patients with schizophrenia (SCZ) and to explore the effect of childhood trauma (CT) on moral cognition in a group of patients with SCZ.

**Methods:**

A total of 99 patients with SCZ and 102 healthy controls (HCs) were included in this study. The Childhood Trauma Questionnaire-Short Form (CTQ) was administered to assess childhood trauma experiences in both groups, while the Moral Identity Measure (MIM) and the Moral Foundations Questionnaire (MFQ) were applied for a comparative evaluation of moral cognition across the two groups. The Positive and Negative Syndrome Scale (PANSS) was administered to assess the psychopathology.

**Results:**

Patients with schizophrenia had significantly greater CTQ scores than HCs (42.77 ± 13.50 vs. 29.11 ± 4.25, *t*=9.697, *p*<0.001). The prevalence of childhood trauma (*χ* ^2^ = 58.452, *p*<0.001) and history of aggressive behaviors (*χ* ^2^ = 23.565, *p*=0.001) among patients with SCZ were greater than that among HCs. In addition, the scores of moral cognition (MIM: 61.82 ± 15.12 vs. 70.88 ± 8.87, *p*=0.001; MFQ: 87.24 ± 22.30 vs. 112.62 ± 23.42, *p*=0.045) in the SCZ group was lower than that in the HC group after controlling for the influence of CT covariates. The MFQ score was negatively correlated with the CTQ score, the emotional abuse (EA) score, the physical abuse (PA) score and the physical neglect (PN) score in SCZ patients. Among HCs, the MFQ score was positively correlated with the CTQ score, as well as with the dimensions of physical abuse (PA) and emotional Neglect (EN). Multiple linear regression analyses revealed that impaired moral cognition performance was significantly predicted by the CTQ score (*beta*=-0.235, *p*=0.034, 95% CI -0.743 to -0.031) in patients with SCZ but was significantly predicted by years of education (*beta*=-0.392, *p*<0.001, 95% CI -4.783 to -1.876), alcohol use (*beta*=0.210, *p*=0.023, 95% CI 2.191 to 29.399) and the CTQ score (*beta*=0.184, *p*=0.046, 95% CI 0.019 to 1.928) in HCs. CTQ moderated the effect of SCZ on MFQ (*B* = 0.516); Simple tests revealed that the group effect on the MFQ was *B*=12.306 at the lower level(-1SD) and *B* = 54.089 at the higher level(+1SD) of the CTQ scores.

**Conclusions:**

SCZ patients exhibit impaired moral cognition. The contribution of CT to the presence of moral cognitive impairments seems to be independent of psychopathology.

## 1 Introduction

Schizophrenia (SCZ) is a chronic and debilitating mental disorder (1, 2) that is often accompanied by various cognitive and social functional impairments (3–7).

The “moral cognition” involves the mental processes related to moral reasoning, judgment, and decision-making (8, 9). Moral cognition is a complex process influenced by a wide range of interpersonal, intrapersonal, social, cultural, and biological factors (10–12). Understanding this multifaceted nature of moral development is important for comprehending the subtleties inherent in ethical decision-making and behavior. Moral Cognitive impairment refers to the deficits in understanding and applying moral principles, assessing the moral value of actions, and making moral decisions. This includes challenges in comprehending moral norms, recognizing moral emotions, understanding, and formulating moral decisions. Moral cognition relies on the proper functioning of various brain regions and neural networks, and is closely related to certain neurocognitive processes, such as executive functions and decision-making (13). Moral cognition is also a part of social cognition (6) and heavily influenced by an individual’s understanding and interpretation of social norms, values, and contextual cues (12).

The functional magnetic resonance imaging (fMRI) offering insights into the neural correlates of moral cognition (14, 15). Compared to nonpsychopaths, psychopaths exhibit decreased activity in the ventromedial prefrontal cortex and anterior temporal cortex when differentiating between moral and nonmoral pictures (15). Individuals with SCZ often present with deficits in executive functions (16), social cognition (17), and emotional processing (7, 18)—key components of the moral cognition framework (19). The prefrontal cortex (20) and temporal lobes (21), regions pivotal for moral reasoning, are notably affected in SCZ, leading to challenges in integrating emotional and cognitive information to make ethical judgments.

Whether patients with SCZ experience moral cognitive impairment has long been a topic of controversy in previous research (20, 22–27). As early as the 19th century, attention was drawn to specific subtypes of schizophrenia (then termed “hebephrenia”) and was described as a disorder associated with difficulties in understanding moral guidelines and proper moral behavior (28). Clinically, some of patients with SCZ exhibit a lack of empathy, superficial emotional experiences, indifference to the well-being of others, and a lack of awareness of their immoral behavior (29). In a study examining moral dilemmas from various perspectives, patients with SCZ took longer to make moral judgements from a third-person perspective, indicating obstacles in moral reasoning (24). This delay in moral reasoning was linked to decreased empathy and a diminished aversion to immoral behavior (24). Additionally, patients with SCZ demonstrated a more utilitarian approach in moral reasoning, which was significantly associated with their high interpersonal conflict (24). Compared with healthy controls (HCs), patients with SCZ showed differences in brain activation patterns during moral judgement in an fMRI study. Specifically, patients with SCZ showed reduced activation in the right hippocampus and increased activity in the superior and inferior frontal gyri (20). These factors may contribute to difficulties in social interaction and moral decision-making, which ultimately impact social functioning.

However, some researchers argue that the evidence to support the claim of a moral cognitive impairment in patients with SCZ is insufficient. In a behavioral study, patients with SCZ and their first-degree relatives made comparable utilitarian decisions in a moral judgement task, with no significant differences compared to HCs (20). Similarly, this conclusion was consistent with a study involving 23 patients with SCZ and 32 HCs, which revealed no significant difference in the tolerance for immoral behavior during moral judgement (23). The juxtaposition of these divergent research outcomes underscores the imperative for further investigations into the domain of moral cognition pertinent to individuals afflicted with schizophrenia.

Childhood trauma (CT) is considered a potential influencing factor that could affect moral cognition in people with SCZ (30). Previous studies have shown that CT not only diminishes emotional bonding between healthy controls (HCs) and their parents (particularly the mother), but also impairs cognitive functions related to moral reasoning, such as theory of mind (ToM) (31), as well as precipitates the decline in cognitive function in late adulthood (32). As suggested in Chemtob’s survival mode theory (33), when people have PTSD, they see even ambiguous situations as threatening, which activates a survival mode process in their brains that results in anger/violence and takes precedence over other, non-violent cognitive functions. Even moral behaviors, such as non-violence in situations that do not warrant it, can be overridden by the brain processing information as threatening due to such trauma history (33). Therefore, CT may play a potential moderator role in the relationship between SCZ and moral reasoning.

The aim of this study was to investigate whether a potential impairment in the moral cognition of patients with schizophrenia (SCZ) exists and to determine whether CT influences moral cognition in patients with SCZ. Accordingly, we propose the following hypotheses: (1) An impairment in the moral cognition of patients with schizophrenia is present, and (2) CT may serves as a moderator or mediator in the relationship between SCZ and the presence of moral cognitive deficits.

## 2 Methods

### 2.1 Participants

From March 2021 through June 2023, a total of 99 inpatients and outpatients with SCZ were recruited from Beijing Anding Hospital, Capital Medical University. Additionally, 102 HCs were enrolled from the local community and matched with the patients on sociodemographic characteristics. The patients were from the “Early Psychosis Cohort Study of Beijing Anding Hospital”, and the HCs were from a study of North China University of Science and Technology. The two studies were approved by the Ethics Committees of Beijing Anding Hospital and North China University of Science and Technology, respectively. All participants in the study provided written informed consent.

The inclusion criteria for patients with schizophrenia were as follows: (1) had a diagnosis of schizophrenia in accordance with the Diagnostic and Statistical Manual of Mental Disorders (DSM-5), which was determined using the Mini International Neuropsychiatric Interview (MINI, 7.0.2) (34); (2) had a minimum of 9 years of education; (3) were native Chinese speakers; and (4) were aged between 13 and 50 years. The exclusion criteria for patients were as follows: (1) had received neuromodulation treatments such as transcranial magnetic stimulation (TMS) or transcranial alternating/direct current stimulation (tACS/tDCS) within the past three months; (2) had ever suffered from another mental illness or serious physical illness; (3) were extremely excited, impulsive, or had risks of self-injury or harming others and were unable to cooperate with the experimenter; and (4) had a history of substance abuse; (5) women who were pregnant or lactating.

The inclusion criteria for healthy controls were as follows: (1) had a minimum of 9 years of education, (2) were native Chinese speakers, (3) were aged between 13 and 50 years. The exclusion criteria were as follows: (1) individuals had a history of any mental illness, substance abuse or a family history of mental disorders as determined by the negative results from the MINI (7.0.2); (2) individuals with severe neurological disorders or medical conditions (such as a history of brain trauma or infection, brain tumor, cerebrovascular diseases, epilepsy, etc.); (3) women who were pregnant or lactating; and (4) individuals who had previously participated in similar studies.

### 2.2 Clinical and neuropsychological assessments

A self-designed questionnaire was used to collect sociodemographic information from participants, including age, sex, years of education, history of aggressive behavior, smoking status, and alcohol consumption.

The Mini International Neuropsychiatric Interview (MINI) was developed to serve as a concise, structured diagnostic interview for screening major psychiatric disorders. It has been extensively employed in diverse environments such as clinical and research settings. Its validity and reliability have been examined through various studies, demonstrating that the MINI bears comparable psychometric properties to other structured diagnostic instruments, with the distinct advantage of requiring a significantly shorter administration time (34).

In addition, basic clinical data such as the duration of illness and antipsychotic usage was also collected from patients’ electronic medical records. The Chinese version of the Positive and Negative Syndrome Scale (PANSS) (35) was administered to assess the severity of psychopathology in patients. The patients’ psychopathology was assessed by the treating psychiatrists using the Positive and Negative Syndrome Scale (PANSS). All the raters have completed the training program to ensure good inter-rater reliability.

#### 2.2.1 Assessment of childhood trauma

The 28-item Childhood Trauma Questionnaire-Short Form (CTQ) was employed for the assessment of CT. The CTQ is a retrospective self-report scale suitable for individuals aged 12 and older (36). The scale utilizes a 5-point Likert scale consisting of “1 = Never”, “2 = Rarely”, “3 = Sometimes”, “4 = Often” and “5 = Always”. The CTQ comprises 5 subscales organized into 2 principal categories (abuse/neglect) and 5 minor categories. The abuse dimension includes emotional abuse (EA), physical abuse (PA), and sexual abuse (SA), while the neglect dimension includes emotional neglect (EN) and physical neglect (PN), with each subscale consisting of 5 items. The scale provides detailed documentation of potentially traumatic events that may have occurred during childhood.

Based on a previous study (37), the specific threshold scores for different dimensions of the CTQ are defined as follows: EA-13, PA-10, SA-8, EN-15, and PN-10. Individuals who reached or exceeded the threshold on any of the CTQ subdimensions were considered to have a history of childhood trauma in the present study.

#### 2.2.2 Assessment of moral cognition

The Moral Identity Measure (MIM) and the Moral Foundations Questionnaire (MFQ) were employed in this study to assess participants’ moral cognition.

The MIM (38) originally consisted of 10 items. However, it has been subsequently revised to incorporate a total of 16 items. The revised MIM utilizes a 5-point Likert scale ranging from 1 (completely disagree) to 5 (completely agree). Higher scores on the MIM indicate a greater level of moral identity among the participants (39).

The original MFQ (40) encompassed five dimensions and a total of 32 items: ① Harm/Care, ② Fairness/Reciprocity, ③ In-group/Loyalty, ④ Authority/Respect, and ⑤ Purity/Sanctity. Each dimension comprises six items, including two validation items designed to evaluate participants’ response accuracy. Considering cultural differences and given that the participants in this study were all Chinese and often considered “Civilization/In-civilization” to be an important indicator of morality, the Chinese scholar Zhao Ying-nan (41) incorporated a “Civilization/In-civilization” dimension based on the relationship between civilization and moral cognition. This dimension consisted of six items and was derived from guidelines provided by the Chinese Civilization website (http://www.wenming.cn/) and the “Beijing Municipal Regulations on the Promotion of Civilized Behavior”. The final Chinese version of the Moral Foundations Questionnaire (MFQ) comprised a total of six dimensions and 38 items. The modified scale displayed good reliability and validity, with an internal consistency coefficient (Cronbach’s α) of 0.93. Additionally, Cronbach’s α for each dimension exceeded 0.6, indicating good internal consistency of the Chinese version of the MFQ. A 6-point Likert scale ranging from 0 (strongly disagree) to 5 (strongly agree) was used to rate the responses on the MFQ. Higher scores on the MFQ indicated a greater degree of moral cognition among the participants.

### 2.3 Statistical analysis

Statistical analyses were performed using SPSS software (version 22.0, SPSS Inc., Chicago, IL, USA). For between-group differences in demographics and scores on the CTQ, MIM, and MFQ between SCZ and HC, categorical data were analyzed using the chi-square test, whereas independent-sample t-tests and analysis of covariance (ANCOVA) were applied for continuous variables. Pearson’s correlation analyses were used to examine the relationships among sociodemographic variables and CTQ, MFQ, and MIM scores if the data met the assumptions; otherwise, point-biserial correlation analysis was performed. Multiple linear regression analyses were conducted to explore the independent predictors of moral cognition in patients with schizophrenia as well as HCs. In the regression analyses, the MFQ scores were entered as the dependent variable, and all variables that showed significant correlations with the MFQ were entered as independent variables. The moderating effect analysis was performed to explore the conditional effects of group membership (Group 1: SCZ; Group 2: HC) on MFQ, moderated by CTQ. The analysis involved a series of regression models to evaluate the main and interactive effects of group and trauma on the outcome variable. The initial model estimated the main effects of group and CTQ on MFQ. Subsequent models included the interaction term (Group × CTQ) to assess the conditional effects. The group and CTQ were centered to reduce multicollinearity and improve the interpretability of the interaction. The results were considered statistically significant if p < 0.05 according to a two-tailed test. Bonferroni adjustment was applied to the exploratory multiple between-group comparisons.

## 3 Results

### 3.1 Sociodemographic characteristics and childhood trauma

No significant differences were observed between the two groups of participants with regard to sex, age, years of education, smoking status or alcohol consumption. However, a significant difference was found between the two groups in terms of a history of aggressive behavior and childhood trauma (see **Table 1**).

**Table 1:** Sociodemographic characteristics and childhood trauma among the two groups of participants ( $\bar{x}$ ± *S*).

Variables	SCZ (n=99)	HC (n=102)	*t/χ* ^2^	*p*
**Sex (Male/%)**	58/58.60	59/57.84	0.011	0.915
**Age (Years)**	37.95 ± 10.07	38.98 ± 11.40	-0.679	0.498
**Years of education**	12.91 ± 3.71	13.48 ± 2.76	-1.241	0.216
**History of aggressive behavior (Yes/%)**	21/21.21	1/0.98	23.565	0.001^*^
**Alcohol use (Yes/%)**	9/9.09	11/10.78	0.125	0.724
**Smoker (Yes/%)**	18/18.18	12/11.76	1.791	0.181
**Duration of illness (months)**	115.34 ± 100.75	–	–	–
**OLZeq (mg)**	15.79 ± 24.43	–	–	–
**PANSS**	64.01 ± 17.15	–	–	–
**Positive**	16.99 ± 6.21	–	–	–
**Negative**	17.00 ± 7.36	–	–	–
**General psychopathology**	30.02 ± 8.24	–	–	–
**CT**	42.77 ± 13.50	29.11 ± 4.25	9.697	<0.001^*^
**EA**	8.30 ± 3.66	5.48 ± 0.95	7.537	<0.001^*^
**PA**	7.04 ± 3.17	5.30 ± 0.91	5.309	<0.001^*^
**SA**	6.55 ± 3.10	5.15 ± 0.48	4.505	<0.001^*^
**EN**	11.67 ± 4.96	7.07 ± 2.61	8.265	<0.001^*^
**PN**	9.21 ± 3.16	6.11 ± 1.68	8.729	<0.001^*^

SCZ, patients with schizophrenia; HC, healthy control; OLZeq, olanzapine equivalent dose of antipsychotics; PANSS, Positive and Negative Syndrome Scale; CT, childhood trauma; EA, emotional abuse; PA, physical abuse; SA, sexual abuse; EN, emotional neglect; PN, physical neglect; *Bonferroni adjustment for the 18 comparisons to a critical α of p<0.0028.

**Figure 1:**
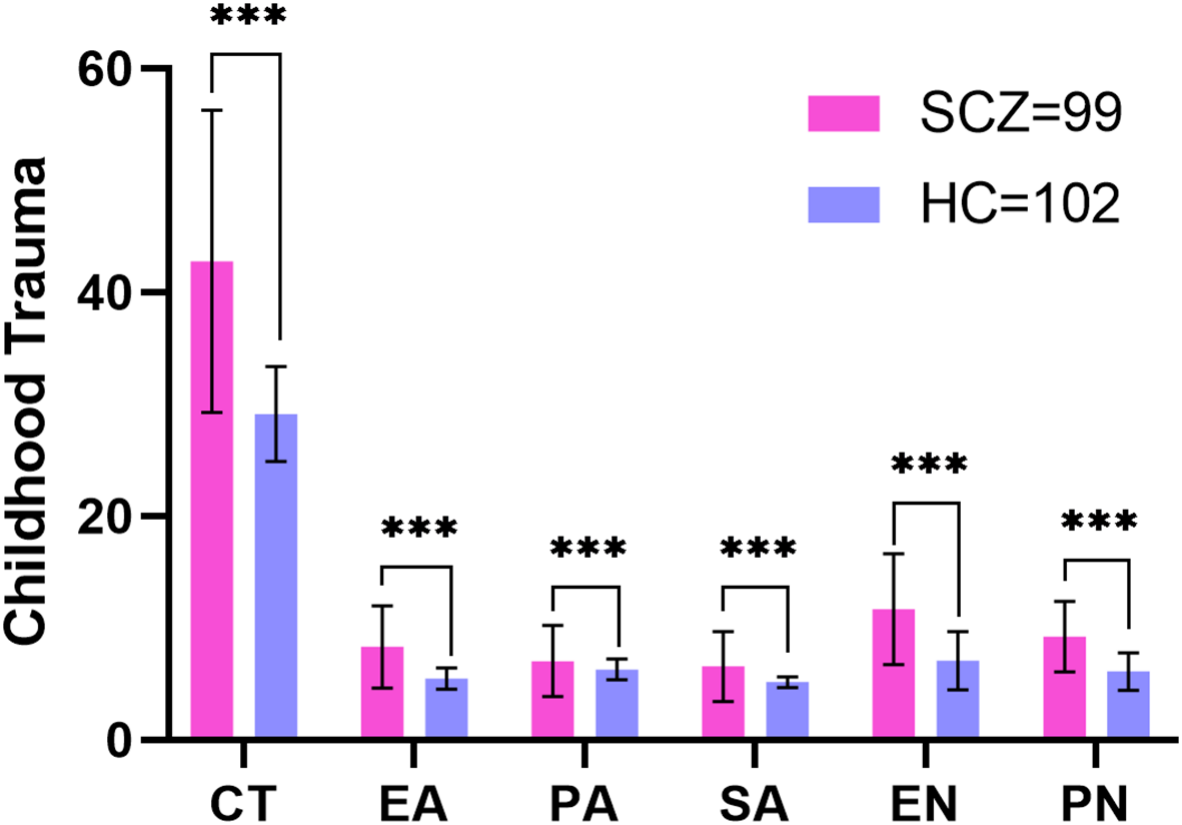
Comparison of childhood trauma in schizophrenia patients and healthy controls. SCZ, patients with schizophrenia; HC, healthy controls; CT, childhood trauma; EA, emotional abuse; PA, physical abuse; SA, sexual abuse; EN, emotional neglect; PN, physical neglect; ***p<0.001.

The CT score of the SCZ group was significantly higher than that of the HC group (*t* = 9.697, *p* < 0.001), as were the scores for each dimension of CTQ, with all *p* values less than 0.001. The percentage of patients with SCZ (53.53%) who experienced CT was significantly greater than that of HCs (3.92%) (*χ* ^2^ = 58.452, *p* < 0.001). Similar results were observed for other dimensions of CTQ, and all *p* values for these dimensions were below the threshold of 0.001 (see **Table 1**; **Figure 1**; **Supplementary Table 1**; **Supplementary Figure 1**).

### 3.2 Moral cognition results

**Figure 2:**
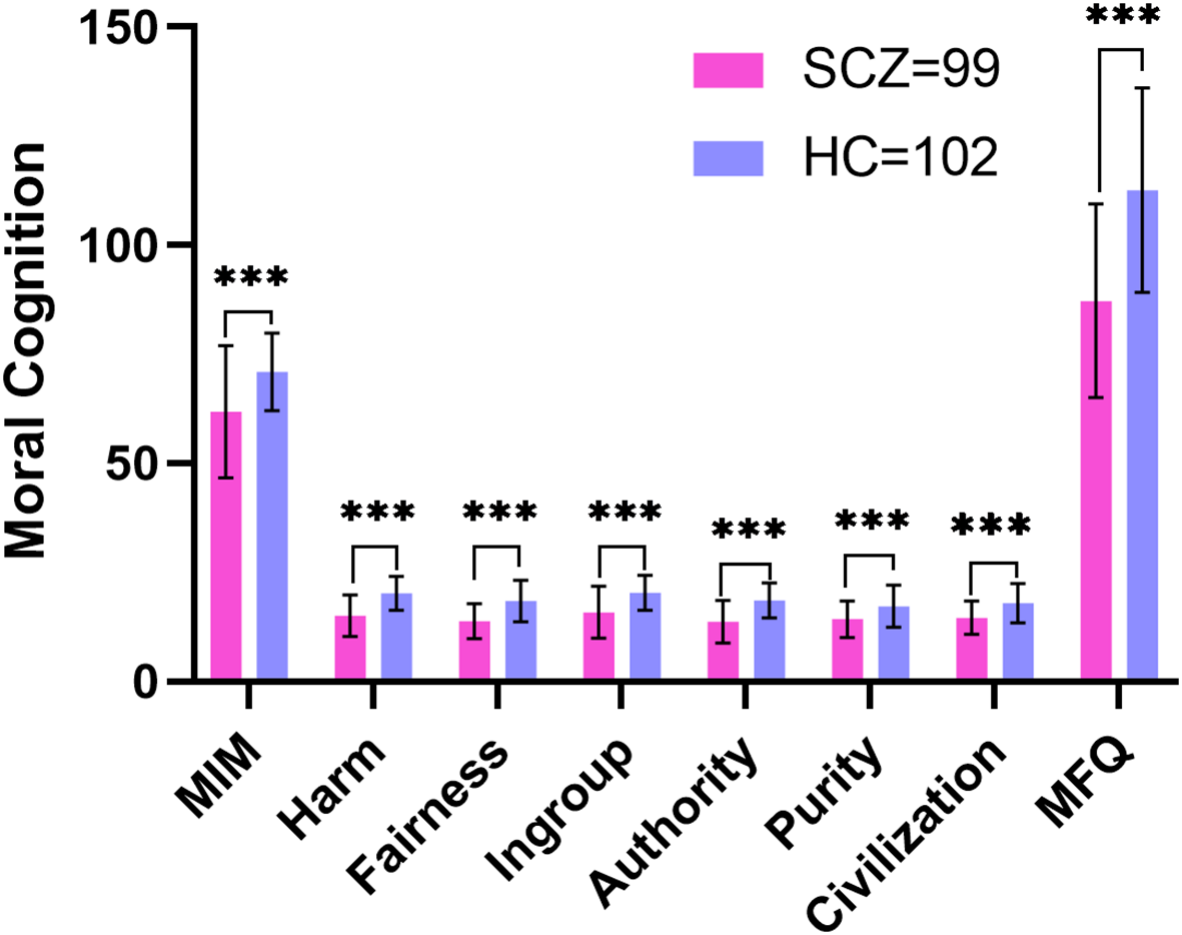
Comparison of the MFQ and MIM scores between patients with schizophrenia and healthy controls. SCZ, patients with schizophrenia; HC, healthy controls; MFQ, Moral Foundations Questionnaire; MIM, Moral Identity Measure; ***p<0.001.

The SCZ group scored lower than the HC group on the MIM (*t*=-5.202, *p*<0.001) as well as on the MFQ (t =-7.862, *p* <0.001) (see **Table 2**; **Figure 2**).

**Table 2:** Comparison of the MFQ and MIM scores between patients with schizophrenia and healthy controls ( $\bar{x}$ ± *S*).

Variables	SCZ (n=99)	HC (n=102)	*t*	*p*
**MIM**	61.82 ± 15.12	70.88 ± 8.87	-5.202	<0.001^*^
**Harm**	15.05 ± 4.72	20.15 ± 3.87	-8.378	<0.001^*^
**Fairness**	13.80 ± 4.00	18.45 ± 4.78	-7.476	<0.001^*^
**In-group**	15.87 ± 5.92	20.31 ± 3.94	-6.281	<0.001^*^
**Authority**	13.64 ± 4.86	18.55 ± 4.04	-7.517	<0.001^*^
**Purity**	14.27 ± 4.21	17.21 ± 4.83	-4.581	<0.001^*^
**Civilization**	14.62 ± 3.80	17.95 ± 4.48	-5.688	<0.001^*^
**MFQ**	87.24 ± 22.30	112.62 ± 23.42	-7.862	<0.001^*^

SCZ, patients with schizophrenia; HC, healthy controls; MIM, Moral Identity Measure; MFQ, Moral Foundations Questionnaire; *Bonferroni adjustment for the 8 comparisons to a critical α of p<0.0063.

### 3.3 Analysis of covariance of moral cognition in patients with schizophrenia

Taking MIM and MFQ scores as dependent variables respectively, the group as the independent variable, and CT as the covariate, ANCOVA was conducted to explore whether differences in moral cognition persisted between the two groups.

The moral cognition of patients with schizophrenia (MIM: 61.82 ± 15.12 vs. 70.88 ± 8.87, *F*= 11.964, *p*=0.001; MFQ: 87.24 ± 22.30 vs. 112.62 ± 23.42, *F*=4.073, *p*=0.045) remained lower than that of HC.

### 3.4 Correlation between childhood trauma and moral cognition

SCZ: MFQ is negatively correlated with CTQ (*r*=-0.247, *p*=0.014), as well as EA (*r*=-0.238, p=0.018), PA (*r*=-0.208, *p*=0.039) and PN (*r*=-0.198, *p*=0.050) in patients with schizophrenia. Scores for the subdimensions of the MFQ had similar negative correlations with scores for the subdimensions of the CTQ, with r values ranging from -0.302 to -0.212 and p values ranging from 0.005 to 0.035.

**Figure 3:**
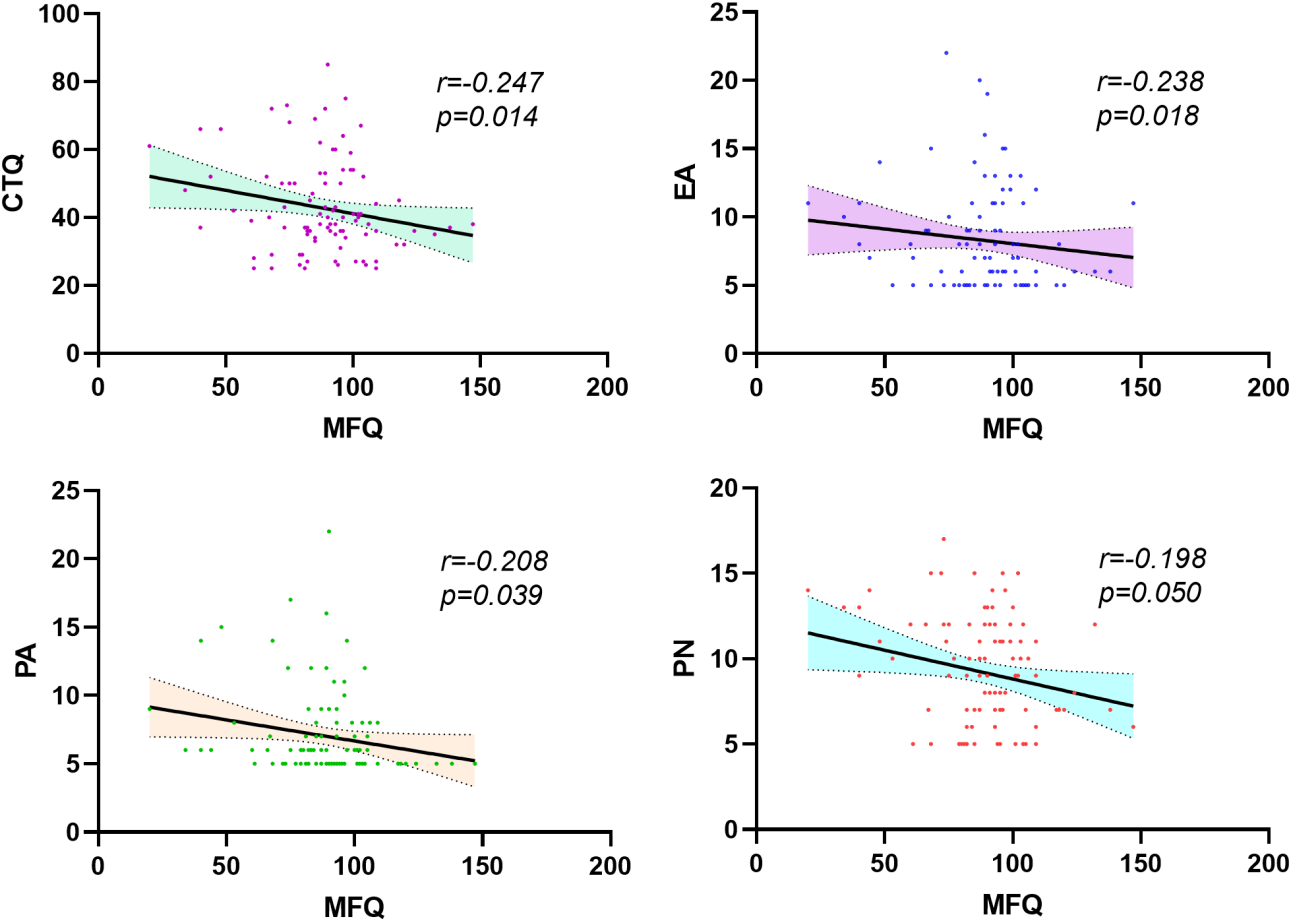
Correlation analysis of MFQ and CTQ scores and their subdimensions in patients with schizophrenia. CTQ, Childhood Trauma Questionnaire; EA, emotional abuse; PA, physical abuse; PN, physical Neglect.

HCs: The MFQ score was significantly positively correlated with the CTQ scores (*r*=0.267, *p*=0.007), as well as with scores for the dimensions of PA (*r*=0.202, *p*=0.042) and EN (*r*=0.203, *p*=0.040) among HCs. The scores for subdimensions of the MFQ were also positively correlated with those of the CTQ, with *r* values ranging from 0.196 to 0.299 and *p* values ranging from 0.002 to 0.049 (see **Figure 3**; **Supplementary Tables 2**, **3**).

### 3.5 Exploring factors that predict moral cognition

SCZ: In the stepwise linear regression analyses, the MFQ score was entered as the dependent variable, and the duration of illness and CTQ scores were entered as independent variables. Higher MFQ score was significantly predicted by the CTQ score (*beta*=-0.235, *p*=0.034, 95% CI -0.743 to -0.031) in SCZ patients.

HCS: In the stepwise linear regression analyses, the MFQ score was entered as the dependent variable, and years of education, alcohol use, and CTQ scores were entered as independent variables. A higher MFQ score was significantly correlated with years of education (*beta*=-0.392, *p <*0.001, 95% CI -4.783 to -1.876), alcohol consumption (*beta*=0.210, *p*=0.023, 95% CI 2.191 to 29.399) and the CTQ score (*beta*=0.184, *p*=0.046, 95% CI 0.019 to 1.928) in HCs (see **Table 3**).

**Table 3:** Results of the stepwise multiple regression analysis of moral cognition.

Variables	Predictor	Beta	*p* value	95% CI
**SCZ (n=99)** MFQ score Adjusted *R* ^2^ = 0.043; *F* (1, 97) =4.679; *p*=0.034				
			
	CTQ score	-0.235	0.034	-0.743, -0.031
**HCs (n=102)** MFQ score Adjusted *R* ^2^ = 0.256; *F* (3, 98) =12.579; *p*<0.001	Years of education	-0.392	<0.001	-4.783, -1.876
	Alcohol consumption	0.210	0.023	2.191, 29.399
	CTQ score	0.184	0.046	0.019, 1.928

SCZ, patients with schizophrenia; HCS, healthy controls; MFQ, Moral Foundations Questionnaire; CTQ, Childhood Trauma Questionnaire-Short Form; EA, emotional abuse; EN, emotional neglect.

### 3.6 Moderating effects of childhood trauma on moral cognition

The model summary indicated a significant fit for the regression model, with *R* ^2^ = 0.288, and *F* = 26.542, *p* < 0.001. The main effects of MFQ predicted by CTQ was not significance (*B* = 0.516, *SE* = 0.266, *t* = 1.939, *p* = 0.054, 95% CI -0.009 to 1.042). In contrast, the main effect of group was significant (*B* = 1.823, *SE* = 0.526, *t* = 3.463, *p* <0.001, 95% CI 0.785 to 2.861).

**Figure 4:**
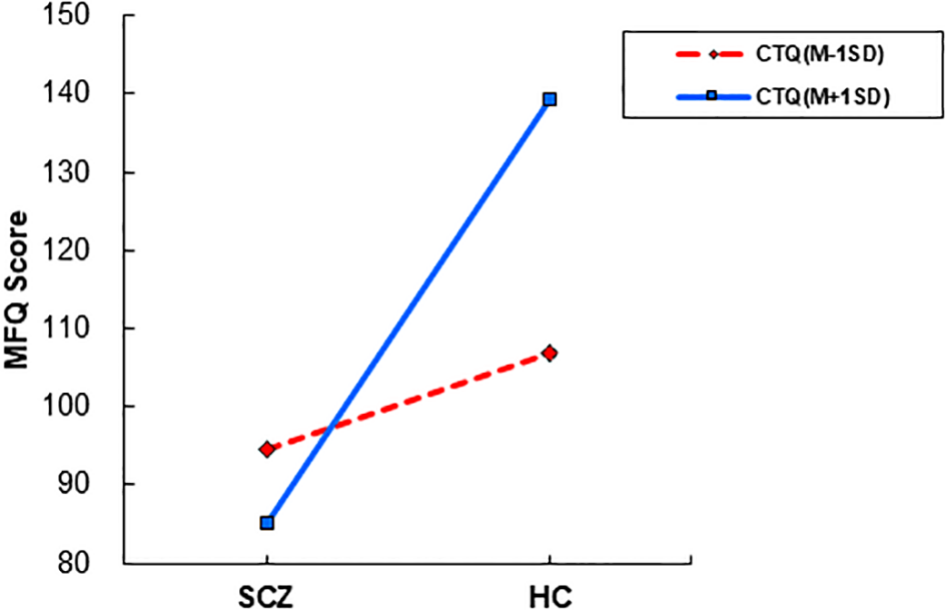
The interactive effects of childhood trauma on moral cognition moderation. SCZ, patients with schizophrenia; MFQ, Moral Foundations Questionnaire.

The interaction between group and CTQ was significant (*B* = 1.823, *SE* = 0.526, *t* = 3.463, *p* = 0.001, 95% CI 0.785 to 2.861), indicating that the effect of group on MFQ varied depending on the level of CTQ. Simple tests revealed that the group effect on the MFQ was *B* =12.306 at the lower level (-1SD) and *B* = 54.089 at the higher level (+1SD) of the CTQ scores; The conditional effects of group at different levels of CTQ are presented in **Figure 4**.

## 4 Discussion

To our knowledge, this is the first study to investigate the effect of CT on moral cognition in schizophrenia patients. The results confirmed our hypotheses. Compared to healthy controls, patients with schizophrenia reported experiencing a higher level of childhood trauma, and were more likely to exhibit moral cognitive impairments. The current study’s results indicated that individuals with schizophrenia experienced a higher rate of childhood trauma compared to their healthy counterparts, which is consistent with previous findings (42–46).

The results of this research confirmed the hypothesis that the moral cognition of patients with schizophrenia is impaired, as evidenced by their lower scores on both the MIM and MFQ and analysis of covariance. As early as the 1980s, research indicated that adolescents with SCZ scored significantly lower on Kohlberg’s Moral Judgement Interview (MJI) than HCs did, and their teachers also reported that the morality of schizophrenia adolescents differed both qualitatively and quantitatively from that of typical adolescents (22). Additionally, in clinical settings, a subset of patients with schizophrenia exhibits a deficiency in empathy, superficial emotional responses, and insufficient awareness of immoral actions (29). These individuals have significant impairments in comprehending moral norms and behaviors, which are specifically characterized by a slowed pace of moral reasoning, a diminished capacity to generate feelings of aversion towards immoral conduct, and a reduction in empathetic abilities (25, 47). Moreover, their behaviors often tend to be egocentric, with a concurrent decrease in prosocial actions (47). Another study involving 45 individuals diagnosed with schizophrenia also reported that this population tends to use stricter evaluation criteria when assessing social transgressions and takes more time in making moral judgements during the process of testing moral permissibility (48). A slower moral judgement is associated with lower empathic abilities, while the tendency to harshly condemn social transgressions is related to poorer perspective-taking and decision-making abilities (48). Research also indicates that in third-person scenarios, patients with schizophrenia tend to exhibit utilitarian characteristics in their moral judgements that differ from those of the general population, and this utilitarian tendency is significantly correlated with their level of interpersonal conflict (24). These findings consistently reveal an impairment in the moral cognition of patients with schizophrenia, which aligns with the results of the present study.

According to Kohlberg’s theory of moral cognitive development (49), the capacity for moral cognition in humans evolves progressively with age. Individuals’ understanding of morality, which encompasses self-awareness, empathy for others, recognition of collective interests, and comprehension of social norms and moral concepts, is subject to ongoing transformation (49). Through continuous learning and imitation, these cognitive processes and experiences are internalized, forming the individual’s own moral standards. Childhood represents a critical period for the development of moral cognition (50). However, childhood trauma, such as physical or sexual abuse, significantly disrupts this normal developmental process. This disruption can have a profound impact on an individual’s moral cognition at these crucial stages.

Bandura’s social learning theory (51) suggests that children, during their moral cognition developmental phase, observe and emulate the behaviors of others, thereby learning and internalizing a spectrum of behavioral patterns that are both directly and indirectly applied to themselves. This encompasses behaviors of a moral transgression, such as physical assault, verbal insult, and sexual abuse. The process of learning and internalization can lead to the formation of detrimental habits in children’s moral cognition, thereby obstructing the natural progression of their moral development. Research indicates that exposure to physical aggression in childhood can lead to a greater likelihood of aggressive behavior in adulthood (52, 53). As the results of the aforementioned studies show, children may learn and adopt violent actions through observation and imitation, which can be both directly experienced and witnessed indirectly with respect to themselves. This learning process can potentially lead to the formation of detrimental moral cognition habits, thereby obstructing the natural progression of moral development.

Furthermore, consistent with Bowlby’s principles on attachment (54), children establish a close attachment relationship with their intimate caregivers, especially their mothers, during childhood that is based on a “trusted” and “secure” “internal working model”, which is crucial for individuals to engage in healthy interactions with others in adulthood. However, childhood trauma disrupts this “internal working model”, leading to difficulties in forming intimate relationships with others. As a result, individuals may manifest psychological defenses and are inclined to exhibit behavior that is deemed ‘immoral’, particularly in an aggressive and violent manner, as a means to manage what they perceive as ‘dangerous’ and ‘unsafe’ interpersonal relationships. As described in Novaco and Chemtob’s survival mode theory (33), when individuals are confronted with danger from the real world or perceive potential threats, such as experiencing severe physical abuse, they tend to process such information in a limited way and significantly increase their alertness, thereby losing the ability to self-monitor. Consequently, they may perceive ambiguous situations as potential threats, even if these situations do not necessitate anger or violent behavior, that is, moral action (i.e., non-violence in situations that do not warrant it) can be overridden by the brain processing information as threatening due to trauma history (33). This result is consistent with the findings in this study that patients with schizophrenia have a greater history of aggressive behavior than healthy controls.

According to revictimization theory (55–58), individuals who have experienced CT, particularly emotional and sexual abuse (55, 57, 58), are more likely to become victims of further victimization or exhibit certain aggressive behaviors. Traumatic experiences in childhood can have a profound impact on the psychological development of individuals, leading to an increased likelihood of experiencing emotional problems such as anxiety, depression, irritability, and posttraumatic stress disorder(PTSD) in adulthood (18, 59, 60). Moreover, one study has shown that CT may increase the tendency to be irritable in patients with schizophrenia, either directly or indirectly, through the misidentification of others’ emotions, which can make them more susceptible to emotional problems and even aggressive behavior (59). Additionally, the pervasive cognitive deficits observed in schizophrenia can be attributed not only to the condition itself but also to compromised decision-making abilities, a prevalent challenge for patients with SCZ that stems from struggles with executive functions and is tied to deficits within the salience network. Both the salience network, responsible for recognizing stimuli that may be of interest or pose a threat, and the frontoparietal network (16), which facilitates attention, decision-making, and action, exhibit impairments in patients with SCZ. The reactivity of the salience network to ambiguous stimuli is heightened by trauma, suggesting a neurological basis for the difficulties that patients with SCZ may experience in responding fittingly to moral dilemmas (33). Consequently, individuals who have suffered CT may be more inclined toward behavior that is considered immoral, a tendency stemming from the complex interplay between emotional dysregulation and impaired social cognition, akin to the negative correlation observed in this study between patients’ childhood trauma and moral cognition.

Some scholars also believe that CT may also affect individual neurodevelopment (42, 61, 62) and have proposed the traumatic neurodevelopmental (TN) model of schizophrenia (63, 64). This model argues that traumatic events have a similar impact on developing brains as do biological abnormalities found in individuals with SCZ (64). The brain inherently connects these physiological alterations to the realm of psychological functioning, underscoring the intricate relationship between brain chemistry and moral cognition (65). Moreover, the TN model also suggests that the abnormal secretion of dopamine, norepinephrine, serotonin, and other neurotransmitters resulting from CT is strongly linked to increased aggression in individuals (66–69).

In our study, we also observed a positive correlation between the moral cognition scores of the HC and their experiences of CT. This correlation may be attributed to the individuals’ enhanced psychological resilience, which allows them to modulate their moral sensitivity in response to adverse childhood events such as trauma or bullying. This hypothesis is supported by an Italian study involving 581 healthy primary school students, which found that children who are victims of bullying and trauma exhibit higher moral sensitivity (70).

In conclusion, SCZ patients exhibit impaired moral cognition. The contribution of CT to the presence of moral cognitive impairments seems to be independent of psychopathology. The findings provide a novel perspective on SCZ and the impact of CT on moral cognitive development in adulthood, laying the groundwork for raising public awareness of CT and reducing stigma with schizophrenia.

## 5 Limitations

Several limitations of this study should be acknowledged. First, the self-assessment and retrospective nature of the questionnaire used to assess moral cognition and childhood trauma experiences may have introduced bias. A combination of self report and behavioral experiments would be a better approaches for the study in the future. Second, the small sample size of our study may have reduced statistical power, affecting the robustness of our findings. Increasing the sample size in future research is essential to confirm our conclusions with greater confidence. Third, the inclusion of both adolescents and adults in our sample may have influenced the results due to potential cognitive differences between these age groups. Future studies should consider the impact of age-related cognitive differences on the outcomes. Fourth, the study primarily focused on the subjective aspect of moral cognition in schizophrenia, which may limit the objectivity compared to those using objective measures such as neuroimaging and clinical electroencephalography. Fifth, moral cognition is a complex process influenced by a wide range of interpersonal, intrapersonal, social, cultural, and biological factors, but it is not feasible to control for all these factors within the current study design.

Last but not least, the study only included schizophrenia patients from a single hospital, which may limit the generalizability of the results due to potential sampling bias. Multicenter studies with larger and more diverse samples are necessary to enhance the validity and applicability of the findings.

## Data Availability

The raw data supporting the conclusions of this article will be made available by the authors, without undue reservation.
