# Supplementary material for: The effect of childhood trauma on moral cognition in patients with schizophrenia
Source: Front Psychiatry. 2024 Sep 30;15:1432407. doi: 10.3389/fpsyt.2024.1432407 (PMC11471594; doi:10.3389/fpsyt.2024.1432407)
Supplement: Supplementary file 1 [file DataSheet1.docx]

**SUPPLEMENTARY MATERIAL**

**Figure**


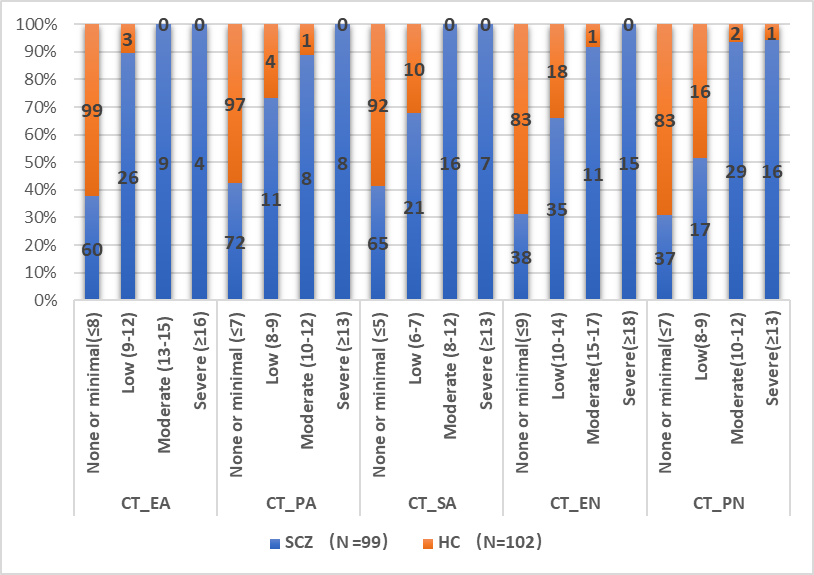


**Supplementary Fig.1** Exposure and difference of childhood trauma in schizophrenia and healthy controls

Abbreviations: SCZ: patients with schizophrenia; HC: healthy controls; CT_EA: Emotional Abuse; CT_PA: Physical Abuse; CT_SA: Sexual Abuse; CT_EN: Emotional Neglect; CT_PN: Physical Neglect

**Tables**

**Supplementary Table 1** Comparison of childhood trauma types and severity between schizophrenia patients and healthy controls

| **Trauma**  **category** | **Classification** | **SCZ**  **(n=99)** | **HC**  **(n=102)** | ***χ*^2^** | ***P*** |
| --- | --- | --- | --- | --- | --- |
| **CT** | **Yes (%)** | 53(53.53%) | 4 (3.92%) | 58.452 | ＜0.001 |
| **EA** | **None or Minimal (≤8)** | 60(60.60%) | 99(97.03%) | 48.553 | ＜0.001 |
|  | **Low to Moderate (9-12)** | 26(26.26%) | 3(2.97%) |  |  |
|  | **Moderate to Severe (13-15)** | 9(9.09%) | 0(0.00%) |  |  |
|  | **Severe to Extreme (≥16)** | 4(4.04%) | 0(0.00%) |  |  |
| **PA** | **None or Minimal (≤7)** | 72(72.72%) | 97(95.09%) | 24.352 | ＜0.001 |
|  | **Low to Moderate (8-9)** | 11(11.11%) | 4(3.91%) |  |  |
|  | **Moderate to Severe (10-12)** | 8(8.08%) | 1(0.98%) |  |  |
|  | **Severe to Extreme (≥13)** | 8(8.08%) | 0(0.00%) |  |  |
| **SA** | **None or Minimal (≤5)** | 65(65.65%) | 92(90.20%) | 40.309 | ＜0.001 |
|  | **Low to Moderate (6-7)** | 21(21.21%) | 10(9.08%) |  |  |
|  | **Moderate to Severe (8-12)** | 16(16.16%) | 0(0.00%) |  |  |
|  | **Severe to Extreme (≥13)** | 7(7.07%) | 0(0.00%) |  |  |
| **EN** | **None or Minimal (≤9)** | 38(38.38%) | 83(81.37%) | 45.487 | ＜0.001 |
|  | **Low to Moderate (10-14)** | 35(35.35%) | 18(17.65%) |  |  |
|  | **Moderate to Severe (15-17)** | 11(11.11%) | 1(0.98%) |  |  |
|  | **Severe to Extreme (≥18)** | 15(15.15%) | 0(0.00%) |  |  |
| **PN** | **None or Minimal (≤7)** | 37(37.37%) | 83(81.37%)) | 54.382 | ＜0.001 |
|  | **Low to Moderate (8-9)** | 17(17.17%) | 16(15,69%) |  |  |
|  | **Moderate to Severe (10-12)** | 29(29.29%) | 2(1.96%) |  |  |
|  | **Severe to Extreme (≥13)** | 16(16.16%) | 1(0.98%) |  |  |

Abbreviations: SCZ: patients with schizophrenia; HC: healthy controls; CT: the total childhood trauma; EA: Emotional Abuse; PA: Physical Abuse; SA: Sexual Abuse; EN: Emotional Neglect; PN: Physical Neglect

**Supplementary Table 2** Analysis of the correlation between demographic, clinical characteristics, childhood trauma, and moral cognition in schizophrenia patients (*r*)

| **Variables** | **MFQ** | **Harm** | **Fairness** | **In-group** | **Authority** | **Purity** | **Civilization** | **MIM** |
| --- | --- | --- | --- | --- | --- | --- | --- | --- |
| **Gender#** | -0.048 | -0.005 | -0.132 | -0.082 | 0.038 | 0.004 | -0.061 | -0.084 |
| **Age** | -0.121 | -0.149 | -0.132 | -0.002 | -0.119 | -0.119 | -0.099 | -0.163 |
| **Years of education** | 0.081 | 0.008 | 0.111 | 0.090 | 0.067 | 0.090 | 0.019 | 0.112 |
| **History of aggressive behavior^#^** | -0.124 | -0.038 | -0.023 | -0.122 | -0.202 | -0.114 | -0.089 | -0.022 |
| **Alcohol^#^** | -0.075 | -0.001 | -0.106 | -0.064 | -0.131 | -0.121 | 0.076 | 0.055 |
| **Smoke^#^** | -0.019 | -0.024 | 0.001 | -0.052 | -0.038 | -0.011 | 0.058 | 0.088 |
| **Duration of illness(months)** | -0.207 | -0.225* | -0.204 | -0.261* | -0.125 | -0.047 | -0.055 | -0.109 |
| **OLZeq (mg)** | 0.616 | 0.813 | 0.794 | 0.435 | 0.167 | 0.622 | 0.733 | 0.958 |
| **PANSS** | -0.108 | -0.102 | -0.161 | -0.092 | -0.092 | -0.017 | -0.061 | -0.104 |
| **Positive** | -0.084 | -0.145 | -0.099 | -0.031 | -0.090 | -0.004 | -0.043 | 0.005 |
| **Negative** | -0.071 | -0.051 | -0.151 | -0.110 | -0.015 | 0.007 | -0.013 | -0.166 |
| **General Psychopathology** | -0.099 | -0.059 | -0.125 | -0.070 | -0.110 | -0.037 | -0.084 | -0.073 |
| **CT** | -0.247* | -0.105 | -0.302** | -0.142 | -0.169 | -0.270** | -0.266** | -0.136 |
| **EA** | -0.238* | -0.126 | -0.279** | -0.187 | -0.129 | -0.225* | -0.238* | -0.069 |
| **PA** | -0.208* | -0.141 | -0.237* | -0.118 | -0.141 | -0.219* | -0.188 | -0.150 |
| **SA** | -0.145 | -0.040 | -0.249* | -0.090 | -0.096 | -0.112 | -0.151 | -0.019 |
| **EN** | -0.149 | -0.011 | -0.153 | -0.081 | -0.108 | -0.212* | -0.197 | -0.105 |
| **PN** | -0.198* | -0.106 | -0.242* | -0.057 | -0.169 | -0.229* | -0.216* | -0.169 |

Abbreviations: #:Point-Biserial Correlation; Gender#: 1: Male; 2: Female; History of aggressive behavior#:1: No; 2: Yes; Alcohol#: 1: No; 2: Yes; Smoke#:1: No; 2: Yes; OLZeq: Olanzapine equivalent dose of antipsychotics; CT: the total childhood trauma; EA: Emotional Abuse; PA: Physical Abuse; SA: Sexual Abuse; EN: Emotional Neglect; PN: Physical Neglect ; MFQ: Moral Foundations Questionnaire; MIM: Moral Identity Measure; *：p＜0.05，**：p＜0.01；***：p＜0.001

**Supplementary Table 3** Analysis of the correlation between demographic, childhood trauma, and moral cognition in healthy controls (*r*)

| **Variables** | **MFQ** | **Harm** | **Fairness** | **In-group** | **Authority** | **Purity** | **Civilization** | **MIM** |
| --- | --- | --- | --- | --- | --- | --- | --- | --- |
| **Gender#** | 0.130 | 0.081 | 0.178 | 0.023 | 0.129 | 0.116 | 0.148 | 0.029 |
| **Age** | 0.041 | -0.027 | 0.107 | 0.011 | 0.011 | 0.041 | 0.060 | -0.080 |
| **Years of education** | -0.419** | -0.290** | -0.363** | -0.370** | -0.417** | -0.369** | -0.417** | -0.017 |
| **History of aggressive behavior^#^** | -0.033 | -0.107 | 0.032 | 0.017 | -0.058 | 0.016 | -0.088 | 0.080 |
| **Alcohol^#^** | 0.308** | 0.208* | 0.260** | 0.359** | 0.223* | 0.386** | 0.203* | -0.035 |
| **Smoke^#^** | 0.069 | -0.006 | 0.081 | 0.072 | 0.093 | 0.022 | 0.100 | 0.039 |
| **CT** | 0.267** | 0.146 | 0.291** | 0.260** | 0.171 | 0.245* | 0.299** | 0.037 |
| **EA** | 0.186 | 0.136 | 0.172 | 0.184 | 0.104 | 0.161 | 0.236* | 0.089 |
| **PA** | 0.202* | 0.187 | 0.182 | 0.147 | 0.156 | 0.137 | 0.271** | 0.063 |
| **SA** | 0.179 | 0.069 | 0.193 | 0.192 | 0.136 | 0.164 | 0.190 | 0.072 |
| **EN** | 0.203* | 0.094 | 0.239* | 0.186 | 0.151 | 0.196* | 0.205* | 0.010 |
| **PN** | 0.122 | 0.042 | 0.144 | 0.158 | 0.033 | 0.130 | 0.136 | -0.023 |

Abbreviations: #:Point-Biserial Correlation; Gender#: 1: Male; 2: Female; History of aggressive behavior#:1: No; 2: Yes; Alcohol#: 1: No; 2: Yes; Smoke#:1: No; 2: Yes; CT: the total childhood trauma; EA: Emotional Abuse; PA: Physical Abuse; SA: Sexual Abuse; EN: Emotional Neglect; PN: Physical Neglect ; MFQ: Moral Foundations Questionnaire; MIM: Moral Identity Measure; *：p＜0.05，**：p＜0.01；***：p＜0.001

**Supplementary Table 4** Comparison of the MFQ and MIM scores between patients with schizophrenia and healthy controls ($\bar{x}\pm S$)

| **Variables** | **SCZ**  **(n=99)** | **HC**  **(n=98)** | ***t*** | ***p*** |
| --- | --- | --- | --- | --- |
| **MIM** | 61.82±15.12 | 70.89±8.84 | -5.133 | <0.001^*^ |
| **Harm** | 15.05±4.72 | 20.14±3.83 | -8.305 | <0.001^*^ |
| **Fairness** | 13.80±4.00 | 18.46±4.80 | -7.402 | <0.001^*^ |
| **In-group** | 15.87±5.92 | 20.28±3.97 | -6.126 | <0.001^*^ |
| **Authority** | 13.64±4.86 | 18.55±4.43 | -7.420 | <0.001^*^ |
| **Purity** | 14.27±4.21 | 17.22±4.88 | -4.546 | <0.001^*^ |
| **Civilization** | 14.62±3.80 | 17.94±4.53 | -5.580 | <0.001^*^ |
| **MFQ** | 87.24±22.30 | 112.59±23.55 | -7.758 | <0.001^*^ |

Abbreviations: SCZ: patients with schizophrenia; HC: healthy controls; MIM: Moral Identity Measure; MFQ: Moral Foundations Questionnaire; *Bonferroni adjustment for the 8 comparisons to a critical α of p<0.0063

**Supplementary Table 5** Results of the stepwise multiple regression analysis of moral cognition

| **Variables** | **Predictor** | **Beta** | ***p* value** | **95% CI** |
| --- | --- | --- | --- | --- |
| **HCs (n=98)**  MFQ score  Adjusted *R*^2^=0.303; *F* (3, 94) = 13.634; *p*＜0.001 | Years of education | -0.385 | ＜0.001 | -4.734, -1.812 |
|  | Alcohol consumption | 0.203 | 0.028 | 1.694, 29.697 |
|  | CTQ score | 0.235 | 0.011 | 0.317, 2.434 |

Abbreviations: HCS: healthy controls; MFQ: Moral Foundations Questionnaire; CTQ: Childhood Trauma Questionnaire-Short Form; EA: emotional abuse; EN: emotional neglect
